# Efficacy and safety of trifluridine/tipiracil plus bevacizumab across different subgroups of patients with refractory colorectal cancer: a meta-analysis

**DOI:** 10.3332/ecancer.2024.1728

**Published:** 2024-07-10

**Authors:** Luís Felipe Leite da Silva, Erick Figueiredo Saldanha, Lucas Diniz da Conceição, Wolney de Andrade Martins, Ronaldo Altenburg Gismondi, Erito Marques de Souza Filho, Renata D’Alpino Peixoto

**Affiliations:** 1 Department of Internal Medicine, Federal Fluminense University, Rio de Janeiro 24070-090, Brazil; 2 Division Medical Oncology and Hematology, Princess Margaret Cancer Centre, University Health Network, Toronto, ON M5G 2M9, Canada; 3 Department of Cardiovascular Sciences, Federal Fluminense University, Rio de Janeiro 24070-090, Brazil; 4 Department of Languages and Technology, Federal Rural University of Rio de Janeiro, Rio de Janeiro 2669 5661, Brazil; 5 Medical Oncology Department, BC Cancer Agency, Vancouver V5Z 4E6, Canada; 6 Instituto Oncoclínicas, Rio de Janeiro 22250-905, Brazil; a https://orcid.org/0009-0004-4397-5200

**Keywords:** colorectal cancer, refractory metastatic colorectal cancer, bevacizumab, trifluridine-tipiracil, meta-analysis

## Abstract

**Introduction:**

Metastatic colorectal cancer (mCRC) patients who are refractory to initial treatment lines exhibit a challenging clinical scenario characterised by a poor prognosis and constrained therapeutic options. This systematic review and meta-analysis assess the integration of bevacizumab into trifluridine-tipiracil (TFD/TPI) therapy for mCRC, examining its benefits across patient subgroups and evaluating safety relative to TFD/TPI monotherapy.

**Materials and methods:**

Following preferred reporting items for systematic reviews and meta-analysis statements, we conducted a thorough literature search from 15 October to 11 November 2023, covering MEDLINE, Embase and the Cochrane database. Data extraction and quality assessment followed Cochrane guidelines, and hazard or odds ratios with 95% confidence intervals (CI) were pooled (*p* < 0.05 significance threshold). The study protocol is registered in PROSPERO (CRD42023484695).

**Results:**

Analysing 770 database results, we included two randomised controlled trials and five observational studies covering over 4,000 patients. Combined therapy exhibited significant improvements in overall survival (OS) hazard ratios (HR 0.60; 95% CI 0.49–0.72; *p* < 0.01) and progression-free survival (HR 0.48; 95% CI 0.40–0.59; *p* < 0.01). Subgroups, including prior bevacizumab exposure (HR 0.70; 95% CI 0.64–0.77; *p* < 0.01) and mutated RAS gene (HR 0.64; 95% CI 0.53–0.77; *p* < 0.01), demonstrated improvements in OSwith bevacizumab.

**Conclusion:**

This meta-analysis underscores the heightened efficacy of TFD/TPI combined with bevacizumab for refractory mCRC compared to TFD/TPI monotherapy across diverse subgroups. Combined therapy has increased grade ≥3 neutropenia and hypertension, while monotherapy is associated with fatigue and anemia.

## Introduction

Colorectal cancer (CRC) is one of the most commonly diagnosed malignancies and is the second leading cause of cancer-related death globally [1, 2]. In the span of the last 20 years, substantial improvement in the understanding of the CRC molecular features and the incorporation of precision oncology has translated to a significant improvement in overall survival (OS) for metastatic CRC (mCRC) [3, 4] patients, with clinical trials reporting a median OS of 30 months [5]. In fact, an increasing number of patients maintain acceptable function status and are eligible to receive a third line of systemic treatment after presenting disease progression following two lines of standard-of-care treatment [6, 7]. However, treatment options for this heterogeneous cohort of patients are limited, and survival outcomes remain poor [8].

Currently, available treatment options for refractory mCRC patients are trifluridine-tipiracil (TFD/TPI) [9], reintroduction or rechallenge of previous treatments such as epidermal growth factor (EGFR) inhibitors plus chemotherapy [10, 11], vascular endothelial growth factor (VEGF) target therapy such as regorafenib [12] and fruquintinib [13]. More recently, studies have reported promising results, revealing a 6-week progression-free survival (PFS) of 42.9% [80% confidence interval (CI) 27.8–59.0] with the addition of the anti-VEGF antibody bevacizumab to TFD/TPI [14, 15]. The same combination was evaluated in phase 3 randomised clinical trials (RCTs) [16] versus TFD/TPI monotherapy, showing improvement in OS of 10.8 versus 7.5 months hazard ratios (HR: 0.61, 95%CI 0.49–0.77) and PFS of 5.6 versus 2.4 months (HR: 0.44, 95%CI 0.36–0.54) [17]. Consequently, the incorporation of bevacizumab into TFD/TPI therapy is currently recommended as the standard of care for these patients [18].

Notably, despite the incorporation of new drugs into the pipeline of advanced CRC treatment, the vast majority of patients with chemotherapy-refractory mCRC will not derive survival benefits. Therefore, we conducted a systematic review and meta-analysis to evaluate the efficacy of TFD/TPI plus bevacizumab compared to TFD/TPI monotherapy for chemotherapy-refractory mCRC, exploring subgroup populations based on RAS mutation status, tumour location, Eastern Cooperative Oncologic Group (ECOG) performance status, previous use of bevacizumab and to address the adverse effects associated with either option.

## Materials and methods

### Eligibility criteria

Studies fulfilling the following criteria were included: (1) RCTs or nonrandomised studies; (2) direct comparison between TFD-TPI monotherapy or its combination with bevacizumab; (3) enrollment of patients with confirmed CRC diagnosis and previous lines of treatment; (4) a minimum follow-up period of 3 months. Only studies presenting extractable data on OS or PFS were considered eligible for inclusion.

Conversely, studies were excluded if they fell under the following categories: (1) limited to abstracts without full-text availability; (2) inefficacy in assessing the intended outcome; and (3) inclusion of patients who had not undergone previous treatment lines. The study protocol is registered in PROSPERO (CRD42023484695).

### Search strategy and data extraction

This systematic review and meta-analysis was performed according to the Preferred Reporting Items for Systematic Reviews and Meta-Analysis Statement (PRISMA) [19]. We systematically searched MEDLINE, Embase and the Cochrane database until 11 October 2023, with the following keywords such as ‘CRC’, ‘TFD/TPI’, ‘bevacizumab’ and ‘TAS-102’. Two authors (LF, LD) independently screened titles, abstracts and full-text publications for study eligibility, with any disagreement resolved by discussion. No restrictions were imposed based on language or publication date.

### Endpoints and subgroup analysis

The efficacy outcomes assessed in this study encompassed OS and PFS. Safety outcomes of interest involved the recording of Common Terminology Criteria for Adverse Events (CTCAE), all-grade adverse events, and grade ≥3 adverse events, which were reported in at least three of the included studies. It is noteworthy that the definitions of PFS and OS remained consistent across all studies included in the analysis.

To explore potential variations among different patient subgroups, we conducted the extraction of results specifically focused on subgroups of interest. Predetermined sub-analyses involved the isolation of data pertaining to (1) RAS mutation status, (2) primary tumour location (PTL), (3) prior administration of bevacizumab and (4) ECOG performance status.

### Quality assessment

Quality assessment was performed for each complete article. For randomised studies, we supplemented the assessment of quality whenever feasible by referencing additional documentation. Nonrandomised studies were evaluated using the ROBINS–1 tool [20] to gauge the risk of bias. The assessment of bias risk in the reviewed randomised studies was conducted by two authors employing the RoB-2 tool [21], as recommended by the Cochrane Collaboration [22] for appraising bias in randomised trials. Bias risk was evaluated for the outcome of OS, guided by the signaling questions of the RoB 2 tool. To explore potential publication bias, we conducted a thorough investigation utilising funnel-plot analysis of point estimates based on study weights and employed Egger’s regression test [23].

### Statistical analysis

HR or odds ratios (OR), accompanied by 95% CI, were derived using the Mantel-Haenszel test [24] and inverse-variance methodologies to compare treatment effects. The DerSimonian and Laird [25] random-effects models were employed to address study heterogeneity. Statistical analyses were performed utilising Review Manager (RevMan) 5.424 [26] and Rstudio version R-4.3.125 [27], with significance set at *p* < 0.05.

Statistical heterogeneity was appraised using the Cochran *Q* test, *I*^2^ statistics and Tau-square via the restricted maximum-likelihood estimator. Inter-study variability was assessed using *I*^2^ metrics and Cochran’s *Q* test, categorising values below 25% or *p* > 0.10 as indicative of low heterogeneity, between 25% and 50% as moderate, and exceeding 50% as high heterogeneity [28]. Multiple sensitivity analyses were executed to scrutinize potential sources of heterogeneity and ascertain the robustness of the primary findings. The leave-one-out method was applied, systematically excluding individual study estimates one by one to gauge their impact on effect-size estimates and heterogeneity. Furthermore, for the primary efficacy outcomes, a fixed-effect meta-analysis was conducted to ensure consistency with the random-effect model. Additionally, a separate pooling of RCTs and observational studies was performed to investigate the potential impact of study design on overall HR.

## Results

### Study characteristics and TFD/TPI plus bevacizumab versus TFD/TPI efficacy

Following the initial search, 770 records were identified. Upon removal of duplicate entries and exclusion of ineligible studies, 43 records underwent thorough assessment against the inclusion and exclusion criteria. Ultimately, 7 studies met the criteria for inclusion in the analysis [16, 17, 29–33], encompassing 4,675 patients included for efficacy analysis, consisting of 2 RCTs and 5 retrospective observational studies. Among these patients, 2,331 (49%) received TFD-TPI in combination with bevacizumab, while 2,344 (51%) were administered TFD-TPI monotherapy (control). The median follow-up ranged from 7.1 to 25.3 months. Although the definitions of interventions and efficacy outcomes were largely consistent across studies, variations were observed in the versions of the CTCAE utilised by some studies. Key characteristics of the main studies are summarised in Table 1.

**Figure 1.**
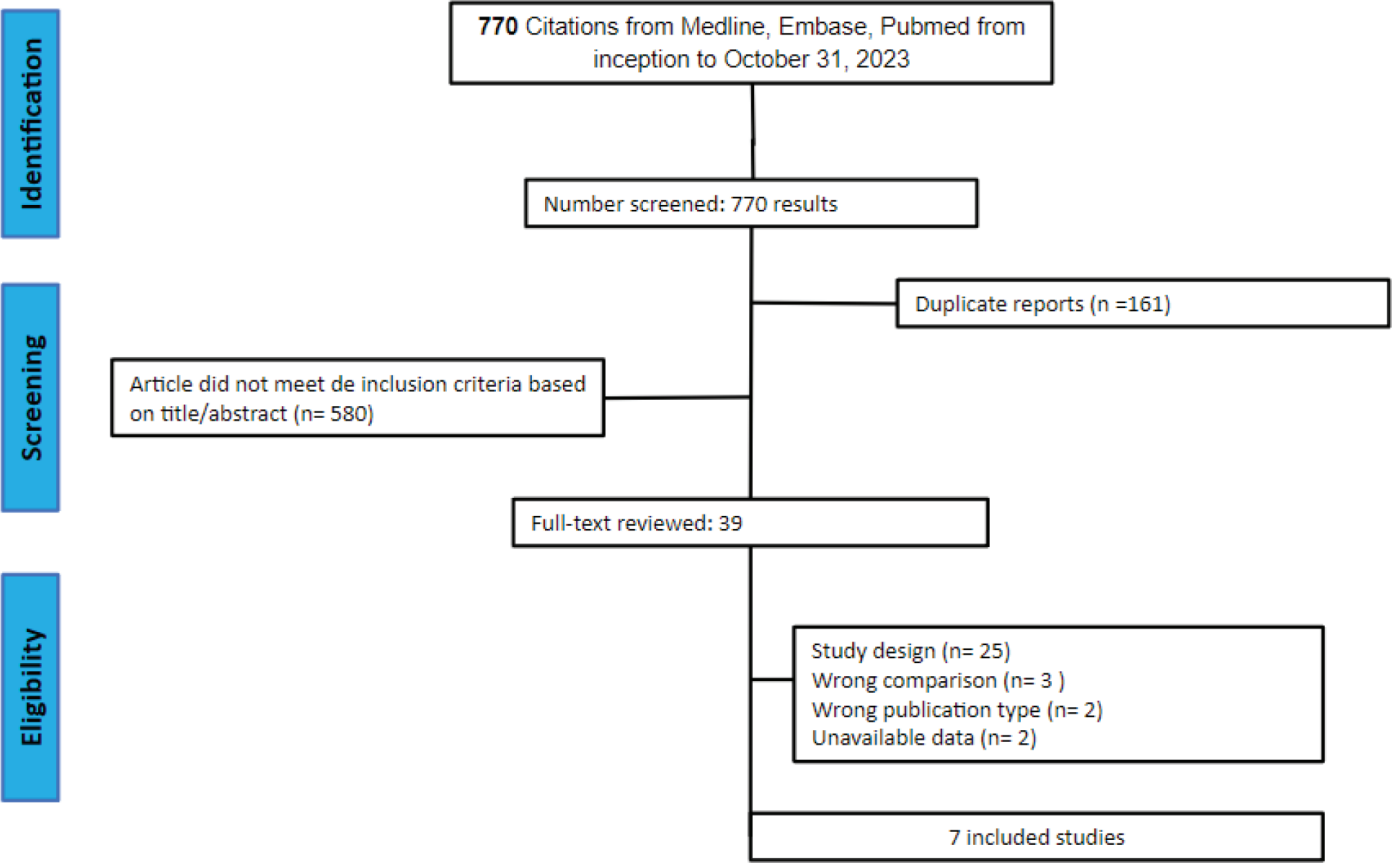
PRISMA flow diagram. PRISMA flow diagram for literature search and selection.

In the cohort receiving TFD/TPI combined with bevacizumab, there was an overall observable trend indicating a notable enhancement in OS (HR 0.60; 95% CI 0.49–0.72; *p* < 0.01; *I*^2^ = 56%; Figure 1A). A sensitivity analysis conducted through iterative exclusion of individual studies revealed that the observed high heterogeneity was predominantly attributed to a single study [33], yet this did not substantially alter the pooled HRs, maintaining a range between 0.57 and 0.67 (Table S1). Similarly, the pooled results for PFS indicated a significant advantage for combined therapy over TFD-TPI monotherapy (HR 0.48; 95% CI 0.40–0.59; *p* < 0.01; *I*^2^ = 45%; Figure 1B). Further sensitivity analysis employing the leave-one-out approach demonstrated minimal fluctuations in the HR upon the exclusion of individual studies.

### Safety profile of TFD/TPI plus bevacizumab versus monotherapy

**Figure 2.**
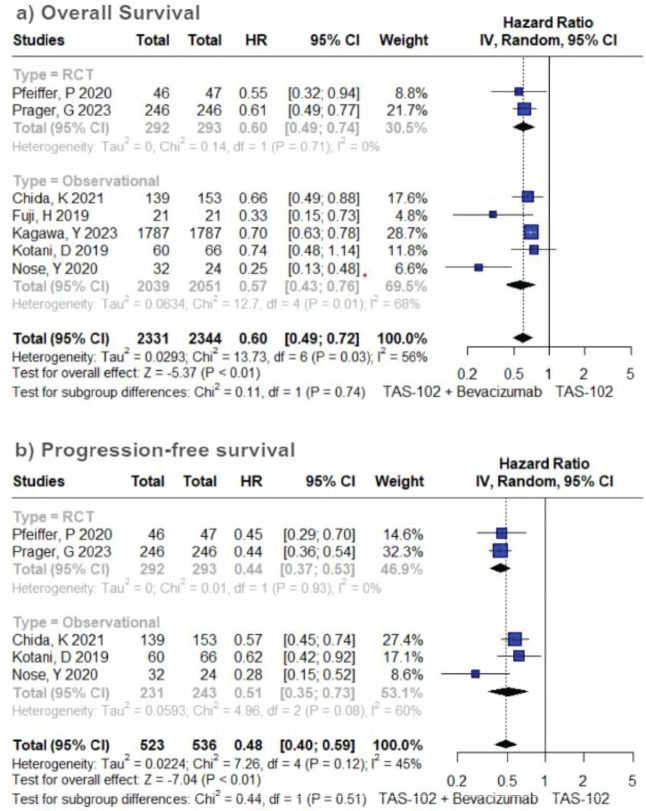
Efficacy analysis of TFD/TPI monotherapy or combined with bevacizumab. Forest plots of the HR of OS and PFS. Squares are the effect size of the individual studies; diamonds, the summarized effect size; horizontal lines, upper and lower border of 95% CI; p-values> 0.05 are considered statistically significant.

In the analysis of Grade ≥3 adverse events (Table 2 and Figure 2A), the incidences of vomiting (OR 0.50; 95% CI 0.12–2.01; *p* = 0.32; *I*^2^ = 0%, Table 3), nausea (OR 1.14; 95% CI 0.39–3.30; *p* = 0.81; *I*^2^ = 0%), diarrhea (OR 0.52; 95% CI 0.11–2.45; *p* = 0.41; *I*^2^ = 30%) and thrombocytopenia (OR 1.69; 95% CI 0.84–3.40; *p* = 0.14; *I*^2^ = 0%) did not exhibit significant differences between the treatment groups. However, there was a notable increase in Grade ≥3 neutropenia with the addition of bevacizumab (OR 1.92; 95% CI 1.08–3.40; *p* = 0.03; *I*^2^ = 72%), while monotherapy showed higher odds of fatigue (OR 0.35; 95% CI 0.15–0.84; *p* = 0.02; *I*^2^ = 0%) and anemia (OR 0.44; 95% CI 0.29–1.66; *p* < 0.01; *I*^2^ = 0%).

For all-grade adverse events, the rates of diarrhea (OR 1.09; 95% CI 0.76–1.55; *p* = 0.65; *I*^2^ = 42%), fatigue (OR 1.12; 95% CI 0.84–1.50; *p* = 0.43; *I*^2^ = 0%), nausea (OR 1.08; 95% CI 0.67–1.75; *p* = 0.74; *I*^2^ = 74%), neutropenia (OR 1.44; 95% CI 0.92–2.26; *p* = 0.10; *I*^2^ = 39%, Table 2) and vomiting (OR 1.28; 95% CI 0.88–1.85; *p* = 0.19; *I*^2^ = 0%) did not significantly differ between the addition of bevacizumab and TFD-TPI monotherapy. Anemia was notably associated with TFD-TPI monotherapy (OR 0.73; 95% CI 0.54–0.99; *p* = 0.04; *I*^2^ = 0%), while thrombocytopenia was linked to the use of bevacizumab (OR 1.72; 95% CI 1.18–2.50; *p* < 0.01; *I*^2^ = 6%).

### Efficacy of TFD/TPI plus bevacizumab across different subgroups of patients

**Figure 3.**
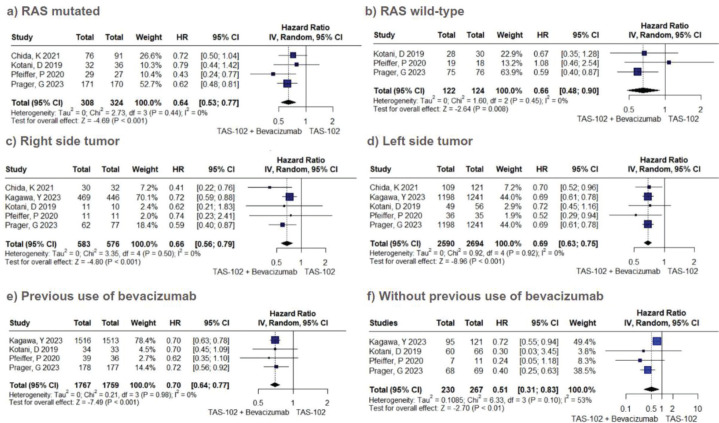
OS subgroup analysis of TFD/TPI monotherapy or combined with bevacizumab. Forest plots of the HR of OS in different subgroups. Squares are the effect size of the individual studies; diamonds, the summarized effect size; horizontal lines, upper and lower border of 95% CI; p-values> 0.05 are considered statistically significant.

In the analysis of OS among patients with mutated RAS status, the use of combined therapy exhibited superiority over TFD-TPI monotherapy (HR 0.64; 95% CI 0.53–0.77; *p* < 0.01; *I*^2^ = 0%, Figure 3A). This trend mirrored the results observed among patients with RAS wild-type status (HR 0.66; 95% CI 0.48–0.90; *p* < 0.01; *I*^2^ = 0%; Figure 3B). Similarly, the advantageous trend of adding bevacizumab was evident in patients with both right-sided primary tumours (HR 0.66; 95% CI 0.56–0.79; *p* < 0.01; *I*^2^ = 0%; Figure 3C) and left-sided tumours (HR 0.69; 95% CI 0.63–0.75; *p* < 0.01; *I*^2^ = 0%; Figure 3D). Moreover, individuals exhibiting a performance status of 0 (HR 0.66; 95% CI 0.54–0.82; *p* < 0.01; *I*^2^ = 0%; Figure 3E), as well as those with a history of prior exposure to bevacizumab (HR 0.70; 95% CI 0.64–0.77; *p* < 0.01; *I*^2^ = 0%; Figure 3F), manifested a statistically significant improvement in OS when subjected to combined therapeutic interventions, albeit to a lesser extent than observed in patients without prior exposure to bevacizumab (HR 0.51; 95% CI 0.31–0.83; *p* < 0.01; *I*^2^ = 53%).

### Quality assessment

The summary of the quality assessment is outlined in Table 1. Three observational studies exhibited disparities in matching intervention and control patients based on critical baseline characteristics, such as previous use of bevacizumab. Moreover, other studies lost two points in the intervention domain classification due to insufficiently detailed reporting of the intervention and control treatment schemes.

**Figure S3.**
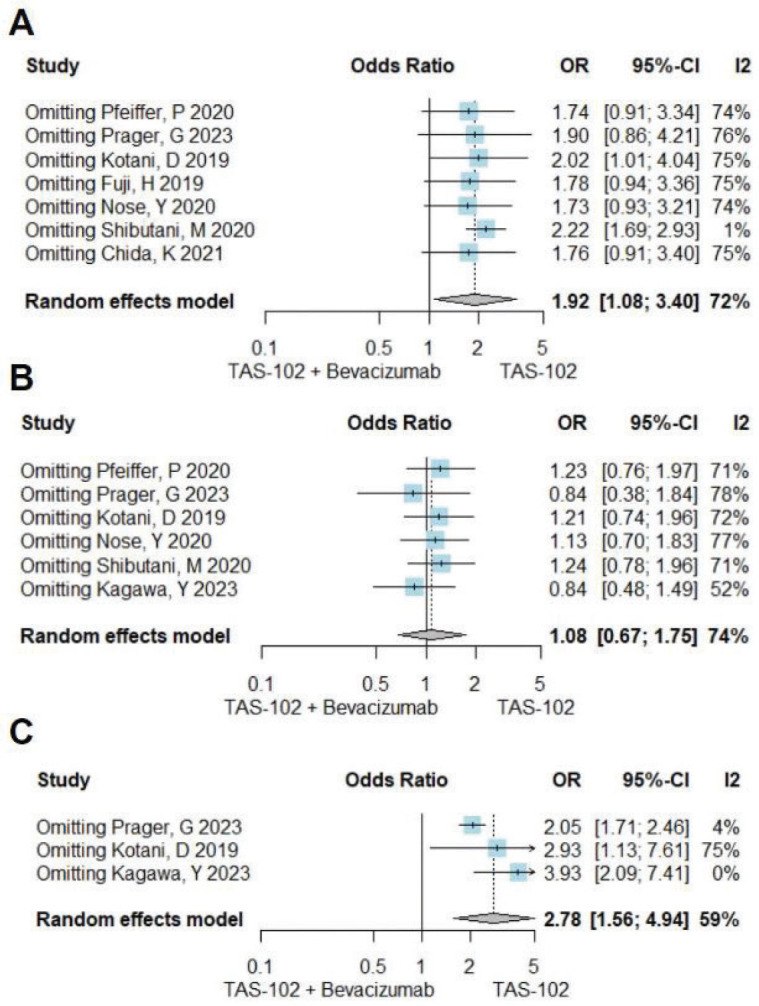
A sensitivity test was conducted for neutropenia - grades 3-4 (Figure A), nausea - all grades (Figure B), and hypertension - all grades (Figure C). In Figure A, upon excluding the study “Shibutani, M 2020,” the I^2^ value reduced to 1%. In Figure C, the omission of “Prager, G 2023” and “Kagawa, Y 2023” resulted in I^2^ values of 4% and 0%, respectively.

Some evidence indicative of publication bias was observed (Figure S3). The funnel plot displayed a symmetrical distribution of studies with similar weights converging toward the pooled treatment effect size as weights increased, except for the study by Nose *et al* [33], which fell outside the funnel plot in the OS analysis. However, Egger’s regression test results indicated no significant evidence of publication bias (*p* = 0.05 for OS and *p* = 0.71 for PFS).

## Discussion

This systematic review and meta-analysis, encompassing over 4,000 patients, compared TFD/TPI in combination with bevacizumab versus TFD/TPI monotherapy in patients with chemotherapy-refractory mCRC. To the best of our knowledge, this is the first study to compare the combination of TFD/TPI with bevacizumab with TFD/TPI monotherapy across pre-specified subgroups of patients. Our study results demonstrated that the combination of TFD/TPI and bevacizumab significantly improved OS and PFS across the entire cohort. Likewise, the superiority of TFD/TPI with bevacizumab was independent of RAS mutational status, PTL as well as previous exposure to bevacizumab, and ECOG performance status. The safety profile of the association TFD/TPI with bevacizumab, when compared to TFD/TPI monotherapy, revealed an increased risk of grade ≥3 neutropenia, whereas monotherapy was associated with an increased risk of anemia.

A better understanding of the CRC molecular features and the incorporation of precision oncology have changed the treatment landscape of advanced CRC. Molecular profiling aids the use of targeted biological agents such as anti-EGFR and anti-VEGF, leading to improvement in OS for mCRC patients [34, 35]. The C-TASK FORCE trial [14], by integrating bevacizumab with TFD/TPI, reported a 16-week PFS rate of 42.9% (80% CI 27.8–59.0). These findings were later replicated in the SUNLIGHT trial, which reported a median OS of 10.8 months with the combination of FTD/TPI with bevacizumab [17] compared to 7.5 with TFD/TPI alone. In a post-hoc analysis from the SUNLIGHT study, the trend of FTD/TPI + bevacizumab benefit in OS was observed irrespective of prior bevacizumab usage [36], which is supported by the pooled analysis of this systematic review.

Our study revealed that TFD/TPI plus bevacizumab improved survival for mCRC patients irrespective of RAS mutational status. The addition of bevacizumab to 5-FU-based chemotherapy has been reported to have limited survival benefits in patients with RAS-mutant CRC, and codon-specific RAS mutations may be prognostic for patients on TFD/TPI treatment [37, 38]. However, findings from the SUNLIGHT trial [19] reported consistent survival benefits of the combination TFD/TPI and bevacizumab, irrespective of RASmutational status, aligned with the results observed in our study. Recently reported a post-hoc analysis of the SUNLIGHT trial revealed the benefit of TFD/TPI plus bevacizumab independently of the KRASG12 mutational status [39], with similar results replicated in recent real-world study [40]. Notably, a recent meta-analysis comparing TFD/TPI versus placebo and/or best supportive care across 2,903 patients also revealed a benefit with TFD/FPI regardless of KRAS mutational status [41].

The prognostic and predictive role of the PTL in advanced CRC is well established in the literature [42, 43]. In a retrospective analysis of the TRIBE trial, which evaluated the intensification of first-line therapy with bevacizumab addition to the FOLFOXIRI regimen, right-sided mCRC had inferior OS than left-sided of 23.7 versus 31.0 months (HR: 1.42, 95%CI 1.09–1.84) [44]. The prognostic role of the PTL has also been evaluated in subsequent lines of mCRC, as reported in a real-world study showing that left-sided tumours had a significant benefit in PFS for patients treated with regorafenib (2.6 versus 1.9 months, *p* < 0.05) [45]. However, the prognostic impact of the tumour sidedness in the chemotherapy-refractory setting for mCRC has scarce evidence. A recent real-world study reported that chemotherapy-refractory CRC patients with left-side demonstrated a survival benefit with TFD/TPI

followed by regorafenib [46]. Our meta-analysis findings suggest that the addition of bevacizumab to TFD/TPI presents clinically significant benefits irrespective of tumour sidedness.

While the addition of bevacizumab has been reported to improve survival outcomes and is approved by both the U.

S. Food and Drug Administration and the European Medicines Agency, considering the incremental costs of such an association can aid in decision-making for regulators [47, 48]. In a study conducted in Japan, TFD/TPI plus bevacizumab had an incremental cost-effectiveness ratio (ICER) of $21,534 per quality-adjusted life-year compared with TFD/TPI monotherapy, indicating a lower threshold than the WHO’s willingness-to-pay recommendations and cost-effectiveness for the Japanese healthcare system [49]. Likewise, Giuliani *et al* [50] evaluated the cost-effectiveness of combining bevacizumab with TFD/TPI, leveraging the ICER and elegantly applying the well-validated European Society for Medical Oncology Magnitude of Clinical Benefit Scale to the Sunlight trial for a comprehensive balance between clinical benefit and cost. The authors concluded that, from the Italian perspective, the combination is cost-effective for the treatment of mCRC patients in the third-line setting. Further studies evaluating the cost-effectiveness of this combination for mCRC patients should be conducted globally, as the prices and access to drugs, such as bevacizumab biosimilars, can differ within healthcare systems across the globe, potentially impacting the adoption of this treatment regimen [6].

Our meta-analysis reported an increase in hypertension with the addition of bevacizumab compared to TFD/TPI monotherapy, reinforcing the need to consider the safety profile of treatment combinations in the later line settings. Bevacizumab cardiotoxicity has been described in multiple studies and a meta-analysis of over 20,000 patients presented the elevated risk of hypertension caused by this targeted therapy [51], whereas TFD/TPI has not demonstrated significant cardiotoxic effects in clinical trials [52]. Furthermore, as reported in a recent real-world analysis [53], our pooled results demonstrated a significant association between TFD/TPI plus bevacizumab and grade ≥3 neutropenia. The meta-analysis conducted by Huang *et al* [41] comparing TFD/TPI with placebo demonstrated an increased risk of adverse events, although there was no significant risk of serious adverse events, results similar to the obtained in our pooled results.

Our study should be considered within the context of its limitations. The heterogeneity observed in the clinical study designs requires caution when interpreting our study results. To address this variability, sensitivity analyses were conducted to assess the robustness of the pooled outcomes. Furthermore, the reliance on study-level data and the absence of access to individual patient data limit the generalisability of our findings.

## Conclusion

In this meta-analysis, we have demonstrated that the addition of bevacizumab to TFD/TPI improved OS for refractory mCRC patients compared to TFD/TPI monotherapy, regardless of RAS mutational status, PTL and prior exposure to bevacizumab. Our study results provide relevant data that can guide patient selection and treatment decisions.

## List of abbreviations

CI, Confidence interval; CPM, Colorectal peritoneal metastasis; CRC, Colorectal cancer; ECOG, Eastern cooperative oncology group; EGF, Epidermal growth factor; HR, Hazard ratio; IQR, Interquartile range; IV, Inverse variance; mCRC, Metastatic colorectal cancer; OR, Odds ratio; OS, Overall survival; PFS, Progression-free survival; RCT, Randomized controlled trial; TFD/TPI, Trifluridine-tipiracil; VEGF, Vascular endothelial growth factor.

## Conflicts of interest

All authors report no relationships that could be construed as a conflict of interest.

## Funding

The authors declare that there was no external funding received for this research. Additionally, the authors declare no financial conflicts of interest related to this work.

## Author contributions

The authors confirm contribution to the paper as follows: study conception and design: LF, ES; data collection: LD, LF; analysis and interpretation of results: LD, EM, RG. Author; draft manuscript preparation: LF, RD, ES, WM. Author. Z. Author. All authors reviewed the results and approved the final version of the manuscript.

## Figures and Tables

**Table S1.** Sensitivity and heterogeneity analyses were conducted for overall survival and progression-free survival. Upon excluding observational studies and Nose, Y 2020, the pooled results indicated low heterogeneity. Furthermore, the combined therapy demonstrated robust results, highlighting its beneficial effects.

	Overall Survival				Progression-free survival		
	n studies	HR (95% CI)	I^2^		n studies	HR (95% CI)	I^2^
**Random effects**	** 7**	**0.65 (0.59; 0.72)**	**56%**		** 5**	**0.48 (0.40; 0.59)**	**45%**
**Fixed effects**	**7**	**0.66 (0.61; 0.72)**	**56%**		**5**	**0.49 (0.42; 0.56)**	**45%**
**Only RCTs**	**2**	**0.60 (0.49; 0.74)**	**0%**		**2**	**0.44 (0.37; 0.53)**	**0%**
**Only observational**	**5**	**0.57 (0.43; 0.76)**	**68%**		**3**	**0.51 (0.35; 0.73)**	**45%**
**Omitting Pfeiffer, P 2020**	**6**	**0.67 (0.61; 0.73)**	**62%**		**4**	**0.49 (0.41; 0.59)**	**58%**
**Omitting Prager, G 2023**	**6**	**0.67 (0.61; 0.74)**	**62%**		**4**	**0.53 (0.44; 0.63)**	**46%**
**Omitting Kotani, D 2019**	**6**	**0.61 (0.52; 0.71)**	**63%**		**4**	**0.47 (0.40; 0.55)**	**46%**
**Omitting Fuji, H 2019**	**6**	**0.67 (0.61; 0.73)**	**53%**		**-**	**-**	**-**
**Omitting Nose, Y 2020**	**6**	**0.67 (0.62; 0.73)**	**1%**		**4**	**0.50 (0.43; 0.59)**	**27%**
** Omitting Chida, K 2021**	**6**	**0.59 (0.49; 0.72)**	**64%**		**4**	**0.45 (0.39; 0.53)**	**38%**
**Omitting Kagawa, Y 2023**	**6**	**0.59 (0.51; 0.69)**	**52%**		**-**	**-**	**-**

**Table S2.**
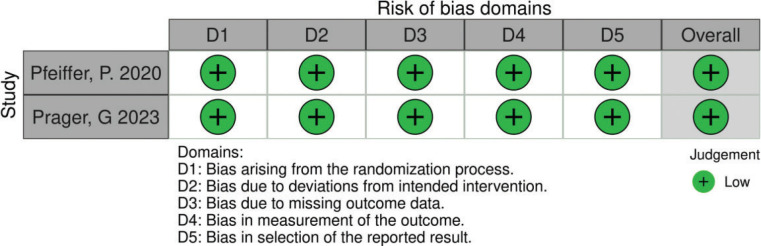
The risk of bias summary displays authors’ judgments regarding each domain of bias for studies utilizing the ROB-2 tool (RCTs). All RCTs demonstrated a low risk of bias.

**Table S3.**
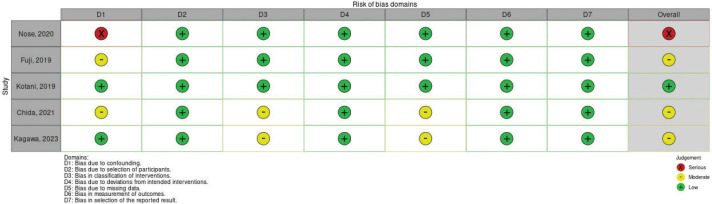
The risk of bias summary presents authors’ assessments of each domain of bias for studies employing ROBINS-I (observational studies). Among the three observational studies (Fuji, 2019; Chida, 2021; Kagawa, 2023), a moderate risk of bias was observed. Conversely, one article (Nose, 2020) exhibited a serious risk of bias.

**Figure S1.**
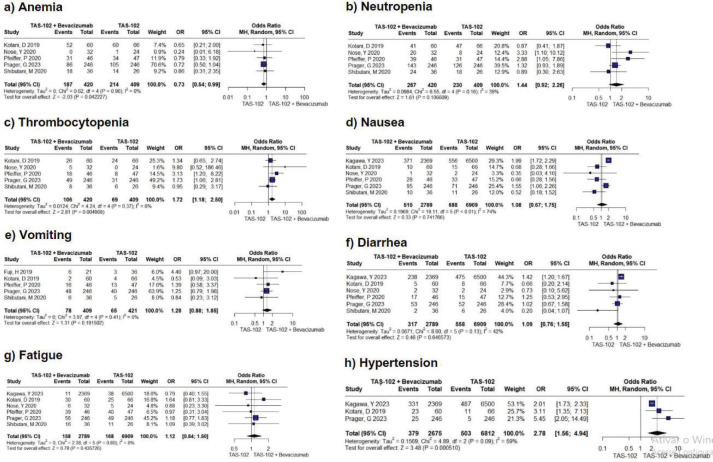
Forest plots depicting adverse events across all grades, including anemia, neutropenia, thrombocytopenia, nausea, vomiting, diarrhea, fatigue, and hypertension, were generated. Notably, anemia, thrombocytopenia, and hypertension exhibited statistical significance with p-values less than 0.05

**Figure S2.**
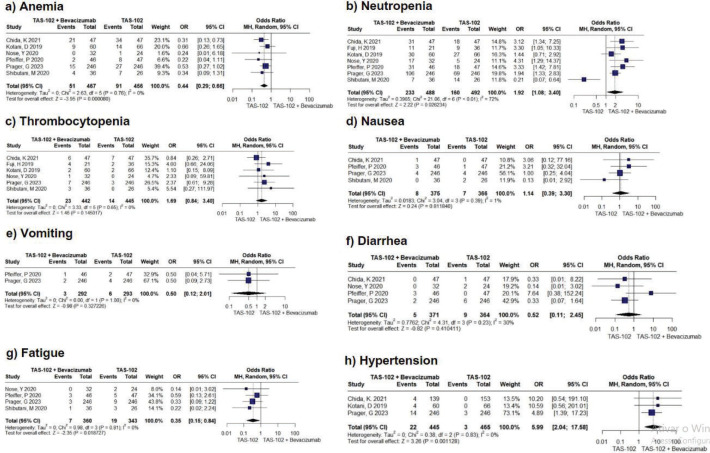
Forest plots for grade ≥ 3 adverse events of anemia, neutropenia, thrombocytopenia, nausea, vomiting, diarrhea, fatigue, and hypertension were generated. Anemia, neutropenia, fatigue, and hypertension did not fall within the null value and showed statistical significance with a p-value less than <0.05.

**Figure S4.**
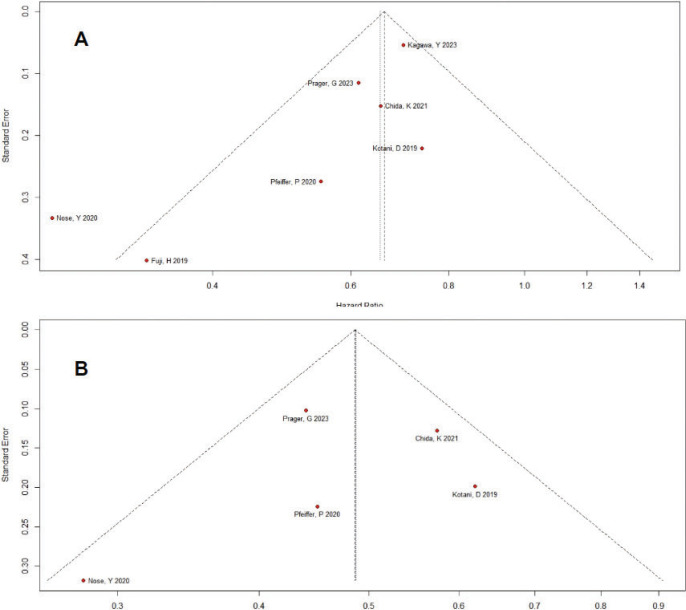
Funnel plot for the outcomes of overall survival (A) and progression-free survival (B). The studies exhibit symmetry around the funnel plot A and B but one study falls outside of the funnel plot A. There might be some concerns of publication bias.

**Table 1.** Study characteristics and patient demographics from included trials.

Baseline characteristics	Prager, G 2023	Pfeiffer, P 2020	Kotani, D 2019	Fuji, H 2019^a^	Nose, Y 2020	Chida, K 2021	Kagawa, Y 2023
**design**	**RCT, multicenter**	**RCT, multicenter**	**Observational**	**Observational**	**Observational**	**Observational**	**Observational**
**Intervention**	**TFD/TPI**	**TFD/TPI**	**TFD/TPI**	**TFD/TPI**	**TFD/TPI**	**TFD/TPI**	**TFD/TPI** *^a^*
**Control**	**TFD/TPI and Bev**	**TFD/TPI and Bev**	**TFD/TPI and Bev**	**TFD/TPI and Bev**	**TFD/TPI and Bev**	**TFD/TPI and Bev**	**TFD/TPI and Bev**
Age, years							
Median	64/62	67/64	65/60	66/67	70/73	65/61	68/68
≥65 (*n*)	117/100	NA	34/19	NA	NA	NA	1,125/1126
Sex							
Female	112/124	17/22	24/25	14/13	9/16	61/52	755/753
Male	134/122	30/24	42/35	7/8	15/16	92/87	1,032/1034
ECOG OS							
0	106/119	NA	42/35	NA	7/12	95/98	NA
1	139/127	NA	21/24	NA	15/19	53/39	NA
No. of metastatic sites							
1 or 2	141/152	NA	52/32	NA	NA	107/90	1,472/1451
≥3	105/94	NA	14/28	NA	NA	46/49	315/336
PTL							
Right	77/62	11/11	10/11	14/14	18/22	32/30	446/469
Left	169/184	36/35	56/49	7/7	6/10	121/109	1,241/1198
RAS status							
Mutaded	170/171	29/27	36/32	9/11	10/17	91/76	NA
Wild-type	76/75	18/19	30/28	12/10	14/14	NA	NA
BRAF status							
Mutaded	11/8	0/2	4/3	NA	NA	7/5	NA
Wild-type	156/159	38/36	52/52	NA	NA	NA	NA
Unknown	79/79	9/8	10/5	NA	NA	NA	NA
Previous treatment lines							
1 or 2	239/240	20/21	35/33	NA	NA	NA	NA
≥3	7/6	27/25	31/27	NA	NA	NA	NA

a Data from the propensity score matching cohort. Baseline characteristics of included studies. Data is presented in the format of patients that received TFD/TPI monotherapy/ TFD/TPI + Bevacizumab; NA: not available; RCT: randomized controlled trial; ECOG PS: Eastern Cooperative Oncology Group performance status

**Table 2.** All grade adverse events of TFD/TPI monotherapy or combined with bevacizumab.

All grade	TFD/TPI + BEV (n)	TFD/TPI (n)	OR, IV, 95% CI	*p* value	*I* ^2^
Anemia	420	409	0.73 (0.54; 0.99)	0.04	0%
Neutropenia	420	409	1.44 (0.92; 2.26)	0.10	39%
Thrombocytopenia	420	409	1.72 (1.18; 2.50)	<0.01	6%
Nausea	515	688	1.08 (0.67; 1.75)	0.74	74%
Vomiting	409	421	1.28 (0.88; 1.85)	0.19	0%
Diarrhea	317	588	1.09 (0.76; 1.55)	0.65	42%
Fatigue	2,789	6,909	1.12 (0.84; 1.50)	0.43	0%
Hypertension	2,675	6,812	2.78 (1.56; 4.94)	<0.01	59%

Pooled results of all grade adverse events. n: number of patients analyzed; IV: inverse variance; CI: confidence interval

**Table 3.** Grade ≥3 adverse events of TFD/TPI monotherapy or combined with bevacizumab.

All grade	TFD/TPI + BEV (n)	TFD/TPI (n)	OR, IV (95% CI)	*p*	*I* ^2^
Anemia	467	456	0.44 (0.29; 0.66)	<0.01	0%
Neutropenia	488	492	1.92 (1.08; 3.40)	0.03	72%
Thrombocytopenia	442	445	1.69 (0.84; 3.40)	0.15	0%
Nausea	375	366	1.14 (0.39; 3.30)	0.81	1%
Vomiting	292	293	0.50 (0.12; 2.01)	0.33	0%
Diarrhea	371	364	0.52 (0.11; 2.45)	0.41	30%
Fatigue	360	343	0.35 (0.15; 0.84)	0.02	0%
Hypertension	445	465	5.99 (2.04; 17.58)	<0.01	0%

Pooled results of grade ≥3 adverse events. n: number of patients analyzed; IV: inverse variance; CI: confidence interval
